# A novel multi-target RNAi adenovirus inhibits hepatoma cell proliferation, migration, and induction of angiogenesis

**DOI:** 10.18632/oncotarget.9531

**Published:** 2016-05-21

**Authors:** Mei Huang, Guangyao Li, Tingting Pan, Ya Cheng, Weihua Ren, Weidong Jia, Jinliang Ma, Geliang Xu

**Affiliations:** ^1^ Anhui Province Key Laboratory of Hepatopancreatobiliary Surgery, Hefei 230001, China; ^2^ Department of Hepatic Surgery, Affiliated Provincial Hospital of Anhui Medical University, Hefei 230001, China

**Keywords:** hepatocellular carcinoma, adenovirus, VEGFR2, CCR1, EpCAM

## Abstract

The pathogenesis of hepatocellular carcinoma (HCC) is a multi-step process involving many genes. Consequently, single gene targeting therapy has limited efficacy, making combination therapy targeting multiple genes a necessity. Based on our previous findings, we constructed a single vector mediating simultaneous expression of multiple short hairpin RNAs (shRNAs) against human vascular endothelial growth factor receptor 2 (VEGFR2), chemokine C-C motif receptor 1 (CCR1), and epithelial cell adhesion molecule (EpCAM), three genes closely related to HCC progression that act through separate pathways. The shRNA vector efficiently downregulated the mRNA and protein of all three molecules in Huh7 hepatoma cells. The vector also inhibited cell proliferation and migration and reduced angiogenesis. Furthermore, this shRNA vector can be recombined into adenovirus, a gene therapy vector, for better *in vivo* application. It thus offers a potentially effective future gene therapy approach to treating human liver cancer.

## INTRODUCTION

Hepatocellular carcinoma (HCC) is the fifth most common malignancy and the third leading cause of cancer-related death worldwide [1, 2]. The pathogenesis of HCC is a very complex multi-step process. Its occurrence, development, and metastasis are closely related with multiple mutations, signaling pathways, and neovascularization [3]. A series of past studies have targeted individual genes dysregulated in HCC both *in vitro* and *in vivo*, but have failed to yield improved therapeutics. More recent tumor gene therapy studies have shifted in focus from single to multiple gene targets. Multiple target gene therapy offers better potential to treat tumor progression, and will become the primary means of tumor gene therapy in the future [4].

Vascular endothelial growth factor (VEGF) is an important angiogenic factor for HCC [5]. The expression of VEGF, as well as its receptor VEGFR2, are extremely low in normal liver tissue, but significantly increased in hepatocellular carcinoma [6]. It has been shown that VEGFR2 is closely related with the growth, invasion, metastasis, and recurrence of HCC. Therefore, inhibition of VEGFR2 expression is an attractive approach for HCC gene therapy.

Many tumor and normal cells produce different chemokines in response to specific stimuli and conditions. Several of these chemokines play crucial roles in tumor progression by recruiting leukocyte infiltration and modulating tumor cell motility. CCR1 mRNA and protein are widely expressed in hepatoma cell lines, and CCL3 and CCR1 are essential to the growth and progression of HCC [7, 8].

Mature liver cells do not express EpCAM under normal conditions, however, Breuhahn et al. reported that 75.9% of chronic alcoholic hepatitis, 63.6% of hepatitis C, and 55.6% of chronic autoimmune hepatitis and hepatitis B patients had liver cells expressing EpCAM, suggesting that EpCAM is related to liver cell regeneration [9].

VEGFR2, CCL1, and EpCAM play important roles in HCC progression through separate signaling pathways. Simultaneous knockdown of these three genes may be more efficient than single gene knockdown in HCC treatment. In our study, we sought to construct a novel RNAi vector simultaneously expressing VEGFR2, CCR1, and EpCAM shRNA cassettes and test its effects on hepatoma cell lines.

## RESULTS

### Expression of VEGFR2, CCR1, and EpCAM in HCC tissues and hepatoma cell lines

**Figure 1:**
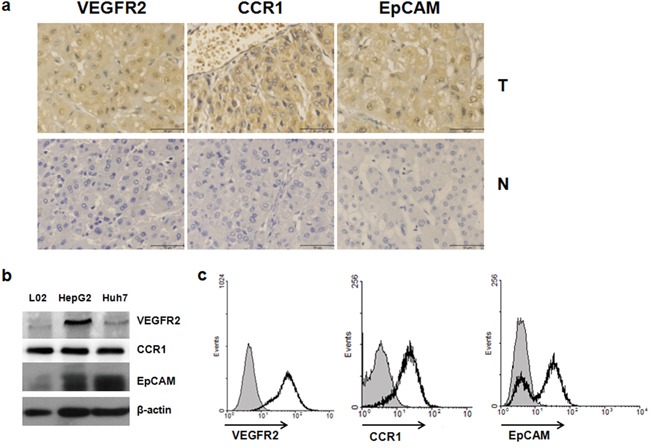
VEGFR2, CCR1 and EpCAM were highly expressed in HCC tissues and hepatoma cell lines **a.** VEGFR2, CCR1, and EpCAM expression of HCC tissues and adjacent non-tumor liver tissue. Localization of VEGF, CCR1, and EpCAM in tissues were determined immunochemically using specific antibodies as described in Materials and Methods. Scale bar, 50 μm; original magnifications, ×400. N, non-tumor liver tissue; T, tumor. **b.** Western blot analysis of VEGFR2, CCR1, and EpCAM expression of hepatoma cell lines. **c.** Flow cytometric analysis of cell-surface VEGFR2, CCR1, and EpCAM expression on Huh7 cells. Open heavy-lined and filled histograms represent those with the test antibody and the control IgG, respectively. The representative results from three independent experiments are shown.

We used immunohistochemistry to detect the expression of VEGFR2, CCR1, and EpCAM in 40 HCC tissues. All three were highly express ed in most HCC samples(38/40, 31/40, and 33/40, respectively, and 28/40, simultaneously), compared with adjacent tissues (Figure 1a). The clinical characteristics of the 40 HCC patients are shown in Supplementary Table 1. There was no significant difference between the expression of VEGFR2, CCR1, or EpCAM and the clinicopathological factors of HCC patients (data not shown). We also analyzed VEGFR2, CCR1, and EpCAM expression in Huh7, HepG2, and L02 cells by Western blot. (Figure 1b), and measured VEGFR2, CCR1, and EpCAM expression on the cell surface of Huh7 cells by FACS (Figure 1c). Both showed that VEGFR2, CCR1, and EpCAM were widely expressed in HCC patient samples and hepatoma cell lines.

### Development of a VEGFR2, CCR1, and EpCAM triple knockdown vector system

**Figure 2:**
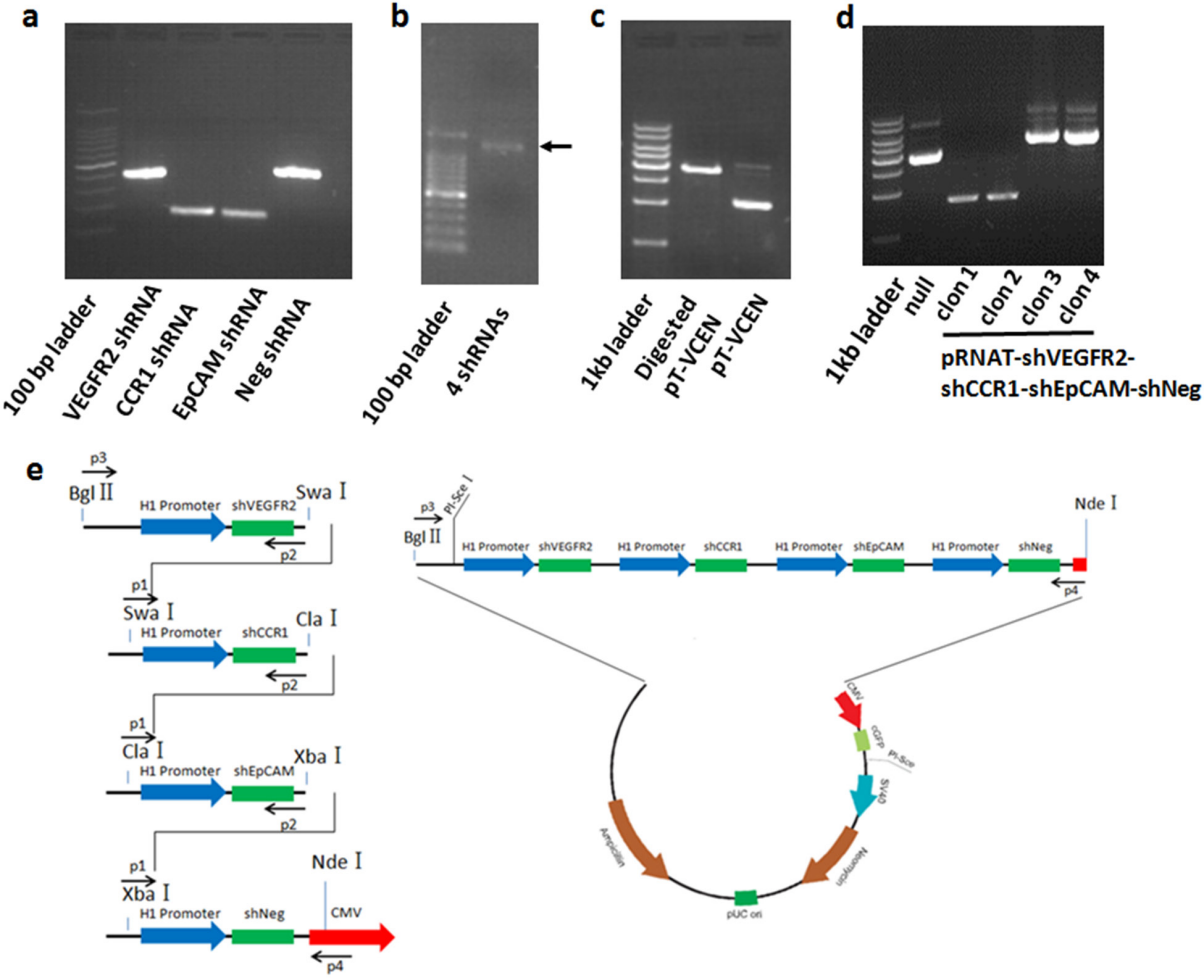
Construction of the multiple target shRNA vector **a.** shRNA transcripts of VEGFR2, CCR1, and EpCAM were amplified from corresponding single shRNA-expressing vectors, including the H1.1 promoter, shRNA-expressing nucleotides, and terminator. More transcript sequences, including the Bgl II restriction site (5,775 bp) to H1.1 promoter site (6,064 bp) and terminator (1 bp) to NdeI restriction site (261 bp), were amplified, respectively, with VEGFR2 and EpCAM shRNA transcript from corresponding single shRNA-expressing vectors for ligation with Bgl II and NdeI double-digested pRNAT-H1.1 vector. **b.** shRNA transcripts of VEGFR2, CCR1, and EpCAM were sequentially ligated, and a large shRNA fragment shVCE (1.4 kb) was obtained. **c.** pRNAT-shVCE was generated by combination of shVCE and pRNAT-H1.1/shuttle via BglII and NdeI restriction enzyme sites. **d.** Clone 3 of pRNAT-shVCE was selected for further study and inserted into a pRNAT-H1.1/shuttle. **e.** A multiple shRNA expressing vector targeting VEGFR2, CCR1, and EpCAM was constructed as shown in the diagram.

To simultaneously silence VEGFR2, CCR1, and EpCAM, a novel vector system was constructed (Figure 2). First, we selected RNAi target sequences for silencing VEGFR2, CCR1, and EpCAM, using siRNA designer software. The shRNA-expressing vectors against the single genes (known as pRNAT-shEpCAM, pRNAT-shVEGFR2, and pRNAT-shCCR1) were constructed by subcloning chemically synthesized oligonucleotides into pRNAT-H1.1/shuttle, a shuttle plasmid for adenovirus. With the constructed shRNA-expressing vectors as PCR templates, the shRNA expression cassettes, including the promoter, RNAi oligonucleotides, and terminator, were amplified together with designed restriction enzyme sites (Figure 2a, Supplementary Figure 1. The shRNA expression sequences of VEGFR2 (400 bp), CCR1 (200 bp), EpCAM (200 bp), and negative control (400 bp) were amplified from corresponding single shRNA-expressing vectors by PCR primers with designed restriction enzyme sites. These shRNA expression cassettes were then sequentially ligated into a large 1.4 kb shRNA fragment shown by the electrophoresis results (known as shVCE), which was further inserted into pRNAT-H1.1/shuttle (Figure 2b, 2c). We confirmed that the clones (including clones 3 and 4) were positively inserted and selected clone 3 of pRNAT-shVCE for further study (Figure 2d). The construction diagram is shown in Figure 2e.

### Knockdown efficiency of the triple knockdown vector system

**Figure 3:**
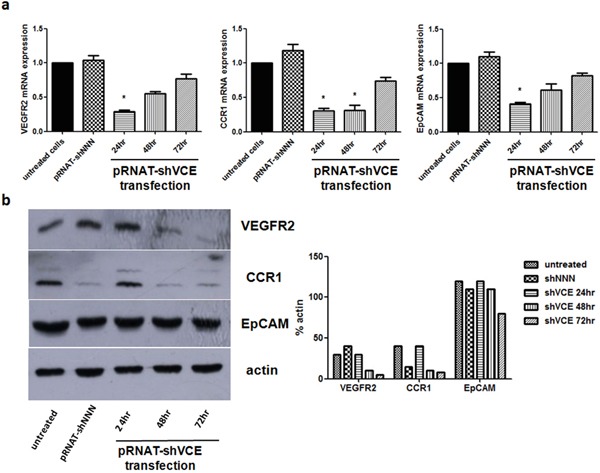
Effect of siRNA of pRNAT-shVCE on VEGFR2, CCR1, and EpCAM expression in Huh7 cells **a.** The mRNA expression levels of VEGFR2, CCR1, and EpCAM were analyzed by quantitative PCR at different times after vector transfection. **b.** Protein expression levels were analyzed by western blot at different times after vector transfection. Statistical analysis for the western blot data is shown on the right. Data are expressed as mean ± SD. * denotes >60% knockdown efficiency versus non-treated Huh7 cells.

To determine the efficiency of the triple RNAi vector for knockdown of VEGFR2, CCR1, and EpCAM, real-time RT-PCR analysis was performed. As shown in Figure 3a, expression levels of VEGFR2, CCR1, and EpCAM were reduced as early as 24 hours after transfection with pRNAT-shVCE compared with that in the pRNAT-shNNN (reported in our previous study) negative silencing group. In addition, the expression levels of VEGFR2, CCR1, and EpCAM proteins were analyzed by western blot. The amount of VEGFR2, CCR1, and EpCAM protein in pRNAT-shVCE transfected Huh7 cells also decreased greatly 24, 48, and 72 hours after infection (Figure 3b).

### Effects of adenovirus-shVCE on cell proliferation and migration

**Figure 4:**
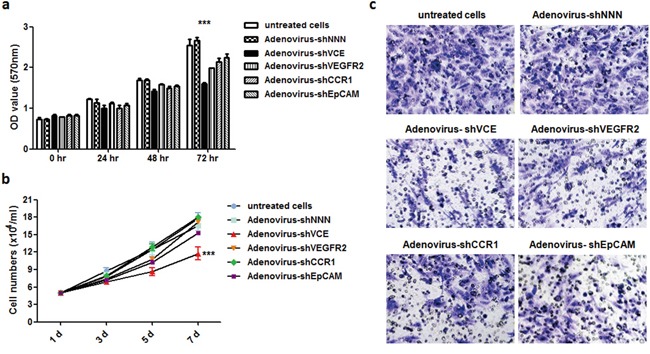
Effect of Adenovirus-shVCE on cell proliferation and invasion Huh7 cells were infected with adenovirus-shVCE, negative silencing Adenovirus-shNNN, or single knockdown adenovirus. **a.** After 0, 24, 48, or 72 hours of incubation, cell viability was determined by MTT assay. **b.** a cell proliferation assay was performed by counting cell number at 1, 3, 5, and 7 days after infection. **c.** Adenovirus-shVCE and other control virus infected Huh7 cell invasion assay. In total, 1 × 10^5^ cells were allowed to invade through Transwell inserts (8 um) coated with Matrigel. The cells on the lower surface of the chambers were stained and imaged. The data are presented as a representative Transwell assay (scale bar, 20 um). (c) *p < 0.05, **p < 0.01,***p < 0.001 versus non-treated cells.

After verification of the high knockdown efficiency of the triple knockdown vector system, we packaged the pRNAT-shVCE plasmids with clinical grade recombinant adenovirus due to its hepatotropism and high infection efficiency [10]. The triple shRNA-expressing vector pRNAT-shVCE, control vector pRNAT-shNNN, and single knockdown vectors pRNAT-shEpCAM, pRNAT-shVEGFR2, pRNAT-shCCR1 were further recombined with the Adeno-X Expression System, forming Adenovirus –shVCE, Adenovirus-shNNN, Adenovirus-shEpCAM, Adenovirus-shVEGFR2, and Adenovirus-shCCR1 (Supplementary Figure 2). We used MTT assay to determine whether Adenovirus-shVCE RNAi had an inhibitory effect on Huh7 cell viability. We found that Adenovirus-shVCE infection of Huh7 cells was associated with a time-dependent inhibition of cell growth, whereas no significant inhibitory effect was observed in Adenovirus-shNNN infected cells, single knockdown adenovirus infected cells, or untreated cells (Figure 4a). Cell proliferation was significantly inhibited at 5 days and 7 days of infection with average proliferation inhibition rates of 28%-32% (Figure 4b). Similarly, cell migration was also inhibited when cells were infected with Adenovirus-shVCE, shown by the transwell assay result (Figure 4c). These data suggest that Adenovirus-shVCE reduces cell proliferation and migration.

### Effect of adenovirus-shVCE on angiogenesis

**Figure 5:**
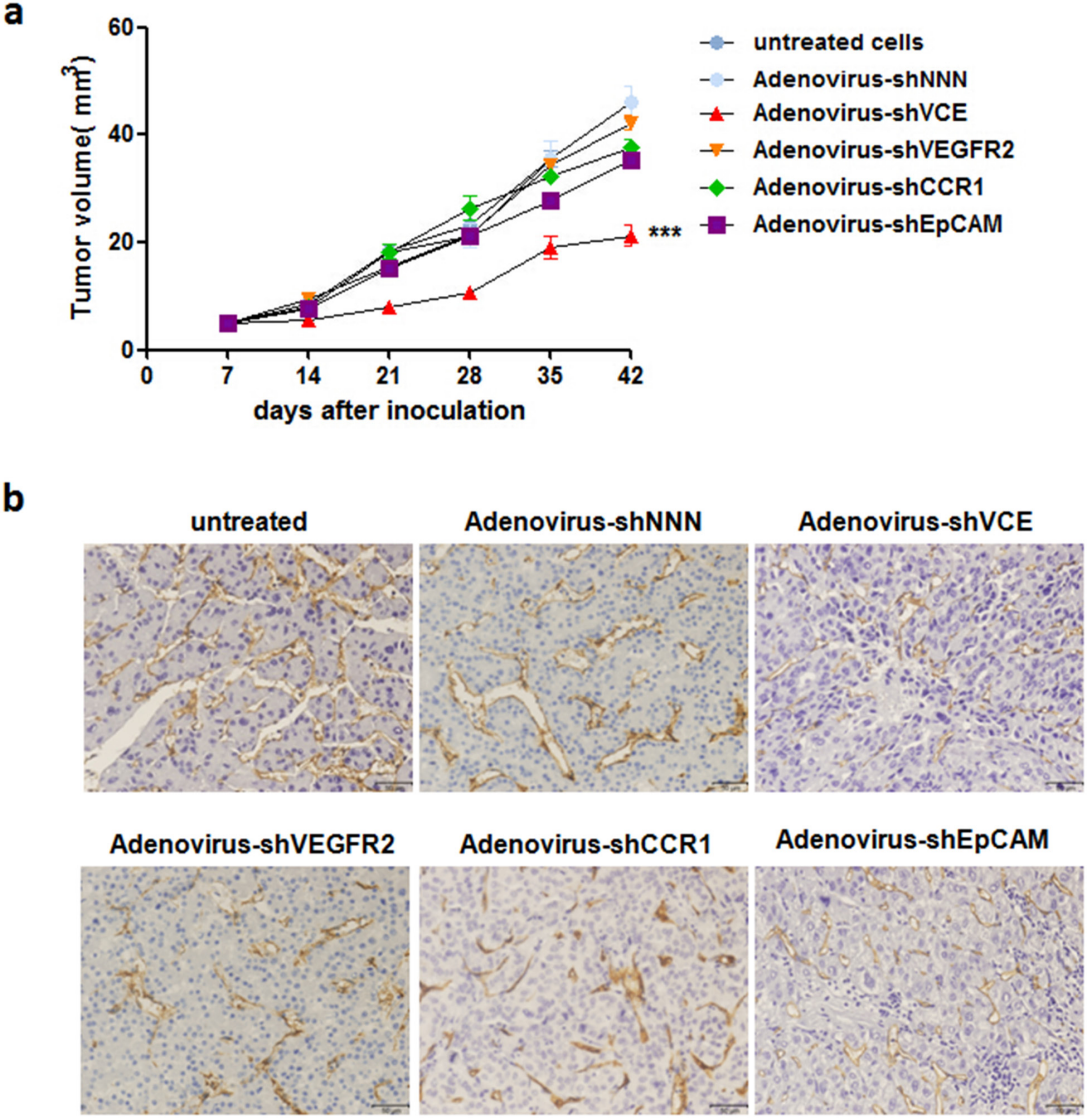
Adenovirus-shVCE inhibited tumor growth and angiogenesis in NOD-SCID mice **a.** Adenovirus-shVCE infected Huh7 cells grew slower than adenovirus-shNNN, adenovirus-shVEGFR2, adenovirus-shCCR1, or adenovirus-shEpCAM infected Huh7 cells in NOD-SCID mice. In total, 2 × 10^6^ Huh7 cells were injected subcutaneously into the backs of mice. The data are presented as mean volumes ± SE; **b.** Angiogenesis was measured with CD34 staining.

To investigate the Adenovirus-shVCE RNAi effect on angiogenesis, we inoculated Adenovirus-shVCE or other adenovirus infected Huh7 cells into NOD-SCID mice. Compared with other adenovirus infected groups, adenovirus-shVCE infected Huh7 cells showed significant inhibition of tumor growth (Figure 5a) and angiogenesis (Figure 5b). Taken together, these results suggest that the multiple target RNAi vector more efficiently repressed cell proliferation, migration, and angiogenesis simultaneously *in vitro* and *in vivo*.

## DISCUSSION

Hepatocellular carcinoma (HCC) is a complex and heterogeneous tumor, with multiple steps in its pathogenesis, including hepatitis and angiogenesis. Despite improvements in diagnosis and treatment, the 5-year postoperative survival rate of HCC patients is only approximately 40% [11, 12]. Biological therapy, primarily gene therapy [13], is increasingly used in combination with liver resection surgery, or chemotherapy [14]. New approaches are urgently needed for the treatment of this prevalent malignancy. Many attempts have been made to target genes in the treatment of HCC, some of them have been effective [15], and some have had no effect. In general, the effect of treatment of any single gene is limited, since HCC involves a number of parallel signaling pathways. Therefore, gene therapy must evolve from single to multiple gene targeting.

We chose VEGFR2, CCR1, and EpCAM as the three target genes because these genes represent angiogenesis, migration, and proliferation, respectively. The novel shRNA vector constructed here was named pRNAT-shVCE (pRNAT-shVEGFR2-shCCR1-shEpCAM-shNeg). Three shRNA expressing cassettes with individual H1.1 promoters and terminators useful for silencing VEGFR2, CCR1 and EpCAM, respectively, were placed in the vector. The pRNAT-shVCE plasmid contains a GFP expression cassette under the CMV promoter, which can be used for image and tracing. This pRNAT-shVCE plasmid can be further combined with pAdeno-X vector to generate recombinant adenovirus, Adenovirus-shVCE, which is more effective *in vivo*.

We found the vector simultaneously and efficiently inhibited mRNA and protein expression of VEGFR2, CCR1, and EpCAM in Huh7 cells. Moreover, inhibition of VEGFR2, CCR1, and EpCAM expression led to decreased cell proliferation, invasion capacity, and angiogenesis in Adenovirus-shVCE infected Huh7 cells. Thus, this study confirms the therapeutic efficacy of combination gene therapy for the progression of HCC, showing that multiple gene therapy may be a more efficient approach for human liver cancer in the future.

In fact, there are many gene candidates for this RNAi system. HCC begins with chronic inflammation of hepatocytes which progressively transform into invasive carcinoma. These events are associated with many molecular abnormalities, including aberrant expression of growth factors and receptors [16]. Targeted agents that inhibit receptor tyrosine kinases, downstream signaling mediators, and angiogenesis have been developed and clinically investigated. Among these targeted agents, Sorafenib, as a multi-kinase inhibitor, has become the standard treatment for advanced HCC [17–19].

Using the construction strategy in the study, we can also simultaneously silence other promising genes for combination gene therapy. Furthermore, more curative effects may be revealed in other malignant tumors.

## MATERIALS AND METHODS

### Patients and tissue samples

Tumor and adjacent non-tumor liver tissue (2 cm away from the tumor edge) were collected for immunohistochemical analysis from 40 patients with HCC who underwent curative surgery between 2012 and 2013 at the Department of Hepatic Surgery, Affiliated Anhui Provincial Hospital of Anhui Medical University, Hefei, China. Informed consent was obtained from each patient, and the study protocol was approved by the Human Research Ethics Committee of Anhui Medical University.

### Western blot

Cells were lysed using RIPA lysis buffer (Beyotime Institute of Biotechnology, China) and protein concentration was subsequently measured using the BCA protein assay. Samples of equal protein concentration were resolved on 10 % SDS-PAGE gels and then transferred to PVDF membranes (Millipore, USA). Membranes were subsequently blocked with 5% non-fat milk and incubated overnight at 4°C with polyclonal rabbit anti-human CCR1 antibody (Boster), polyclonal rabbit anti-human VEGFR2 antibody (Boster), or mouse anti-human EpCAM antibody (Boster). Membranes were then washed with Tris-buffered saline/0.1 % Tween three times and probed with horseradish peroxidase (HRP)-conjugated secondary antibody for 2 h at room temperature. Immunoreactive bands were visualized using the enhanced chemiluminescence (ECL; Pierce, USA) kit. Band density was measured using ImageJ Software (National Institutes of Health, USA). β–actin expression served as the internal control.

### Cell culture

Human hepatoma-derived cell lines including Huh7 (differentiated hepatoma cell line), HepG2 (epithelial-like cell line) cells, and L02 cells were maintained in Dulbecco's modified essential medium (DMEM) supplemented with 10% heat-inactivated fetal bovine serum in a humidified incubator at 37°C in 5% CO_2_.

### Flow cytometric analysis

Cultured Huh7 cells were harvested and incubated in a washing buffer containing 5% normal goat serum to block nonspecific binding. For antibody staining, 5 - 10 × 10^5^ cells were incubated with fluorescein labeled antibody. The monoclonal antibodies used for flow cytometry in this study included PE mouse anti-human CD309 (VEGFR-2, FLK1); Alexa Fluor^®^ 647 Mouse anti-Human CD191 (CCR1); PE mouse anti-EpCAM. Stained cells were analyzed by FACSCalibur, and data were analyzed using WinMDI2.9 software.

### Immunohistochemical analysis

Four micron-thick tissue sections were deparaffinized in xylene, rehydrated, subjected to microwave antigen retrieval in citrate buffer (10mM, PH 6.0) for 20 min, and then cooled at room temperature. Endogenous peroxidase activity was quenched by soaking in 3% hydrogen peroxide. Sections were then blocked with normal goat serum at room temperature to prevent non-specific binding. Subsequently, individual sections were incubated overnight at 4°C with rabbit anti human VEGFR-2 polyclonal antibody (Zhongshan Golden Bridge Biotechnology Inc), goat anti human CCR1 polyclonal antibody or rabbit anti human EpCAM polyclonal antibody (Abcam). After rinsing in phosphate-buffered saline (PBS), the sections were incubated for 30 min with HRP-conjugated secondary antibody (Zhongshan Golden Bridge Biotechnology Inc). After subsequent washing, immunoreactivity was visualized with the chromogen 3,3′-diaminobenzidine. Finally, all slides were counterstained with hematoxylin, dehydrated, and mounted.

### Preparation of shRNA expressing vectors simultaneously targeting human VEGF, CCR1, and EpCAM

We constructed the multiple shRNA expressing vectors against human VEGF, CCR1, and EpCAM as described previously [10]. The chosen siRNA target sequences are as follows: VEGFR-2, 5′-GGT CCA TTT CAA ATC TCA ACG-3′; CCR1, 5′-GCT TCC ATG CCA GGC TTA TAC-3′; EpCAM, 5′-GCT GGC CGT AAA CTG CTT TGT-3′; and negative silencing control sequence, 5′-GAG ACC CTA TCC GTG ATT A-3′. The shRNA expressing cassettes, including the H1.1 promoter and corresponding oligonucleotides, were amplified from the single shRNA-expressing vector, using the primers listed in Supplementary Figure 1. Further ligation was performed via the same restriction enzyme site to construct the multiple shRNA-expressing vectors. The viruses were propagated in HEK293 cells and purified by CsCl discontinued density gradient centrifugation. Viral titers were determined with Adeno-X Rapid Titer Kit (BD Biosciences Clontech).

### Cell viability and proliferation assay

Cell viability was assessed by methyl thiazol tetrazolium (MTT) assay. Cells were plated in 96-well plates containing 10% FBS. After infection with Adenovirus-shVCE, control virus Adenovirus-shNNN, or single knockdown virus (Adenovirus-shEpCAM, Adenovirus-shVEGFR2, or Adenovirus-shCCR1) for 0, 24, 48, or 72 hours, cells from each group were collected and plated in 96-well plates at a density of 1.0 × 10^4^ cells/well for MTT assay. Untreated cells served as control. Absorbance was measured at 570 nm. Each assay was performed in triplicate. Cell growth (mean absorbance ± standard deviation) was plotted versus time. A cell proliferation assay was performed by counting cell number. Cells were plated at a density of 5.0 × 10^4^ cells/mL in 24-well plates and infected with Adenovirus. Cells were harvested daily and counted.

### Cell invasion assay

Transwell invasion assays were performed using transwell chambers with 8 μm pore size filter membranes (Millipore). Cells were seeded into the upper chamber and grown in 600 μL DMEM containing 10% FBS loaded in the lower chamber. Then, the transwell chambers (Corning Costar, NY, USA) were incubated in a 37°C, 5% CO_2_, humidified incubator for 24 hr. The cells on the inner surface of the filter membrane were removed with a cotton swab. Cells on the lower surface of the membrane were stained with 0.5% toluidine in 2% Na_2_CO_3_ and counted in five random fields by 200x magnification light microscopy (Olympus, Beijing, China).

### Animals

Five-week-old male NOD-SCID mice were purchased from Shanghai Laboratory Animal Center, Chinese Academy of Science (Shanghai, China). All mice were housed in a specific pathogen-free, temperature-controlled microenvironment with 12-hour day-night cycles. All procedures were in compliance with the regulations of animal care of Anhui medical university.

### Mouse models of tumor growth and angiogenesis

Adenovirus-shVCE or control virus infected Huh7 cells (2 × 10^6^ cells per tumor) were harvested and resuspended in 100 μL of PBS. The cells were injected subcutaneously into the backs of 5-wk-old NOD-SCID mice. Tumor volume (TV) was calculated using the following formula: TV = W^2^ × L × 0.5. For angiogenesis analysis, CD34 staining was performed on tumor sections.

### Statistical analysis

Data are expressed as mean ± SD and processed by the statistical analysis software GraphPad Prism 5. Comparisons among all groups were performed with unpaired Student's t-test. P value of less than 0.05 was considered significant. The results shown in each of the figures in this paper are representative of at least three independent experiments.

## SUPPLEMENTARY MATERIALS FIGURES




