# Supplementary material for: A novel multi-target RNAi adenovirus inhibits hepatoma cell proliferation, migration, and induction of angiogenesis
Source: Oncotarget. 2016 May 21;7(36):57705–13. doi: 10.18632/oncotarget.9531 (PMC5295383; doi:10.18632/oncotarget.9531)
Supplement: Supplementary file 1 [file oncotarget-07-57705-s001.pdf]

SUPPLEMENTARY MATERIALS

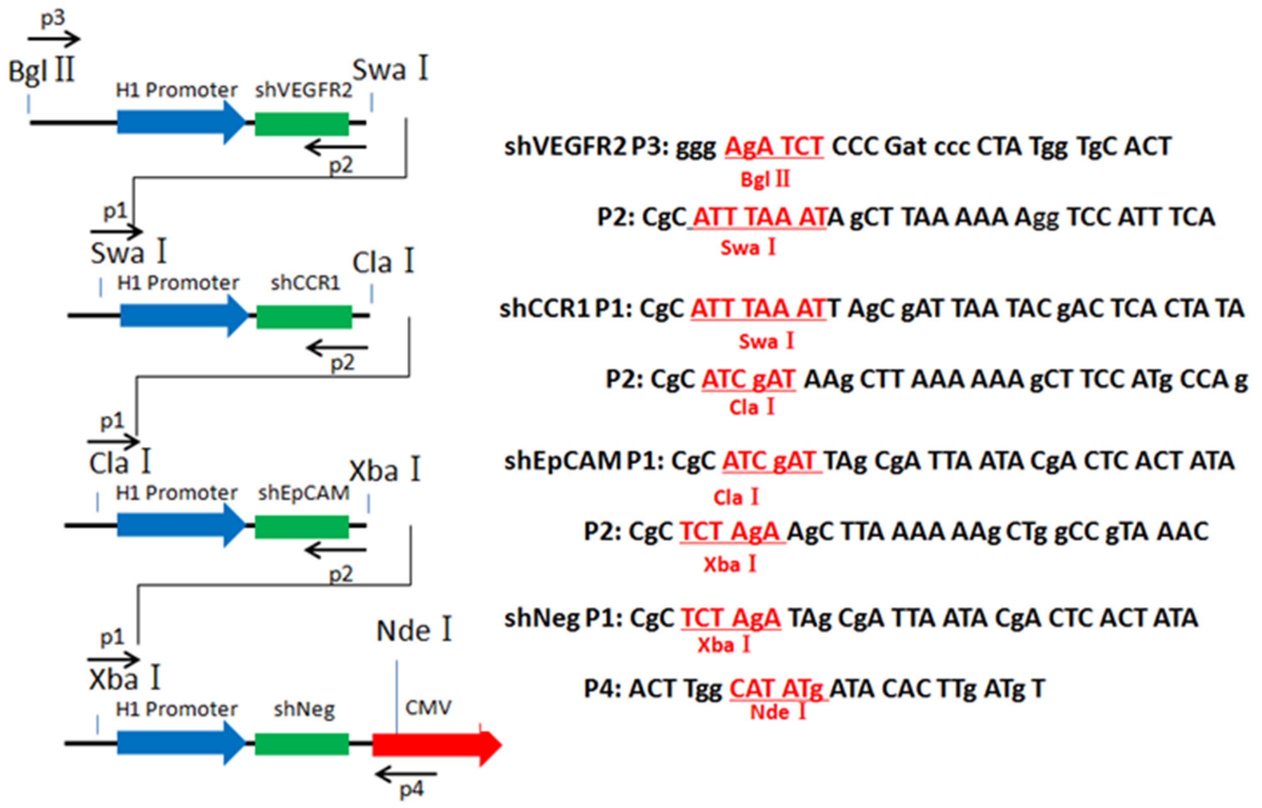

Supplementary Figure S1: Schematic representation of triple shRNA expression vector against VEGFR2, CCR1 and EpCAM pRNAT-VCE generated in the study. The P2 of shVEGFR2 has the same enzyme site, Swa I, as P1 of shCCR1; the P2 of shCCR1 has the same enzyme site, Cla I, as P1 of shEpCAM; the P2 of shEpCAM has the same enzyme site, Xba I, as P1 of shNeg; the P1 of shVEGFR2 and the P4 of shNeg have the same enzyme site, Bgl II and Nde I as pRNAT-H1.1/shuttle. Diverse shRNAs were ligated with the same enzyme site and then subcloned into pRNAT-H1.1/shuttle.

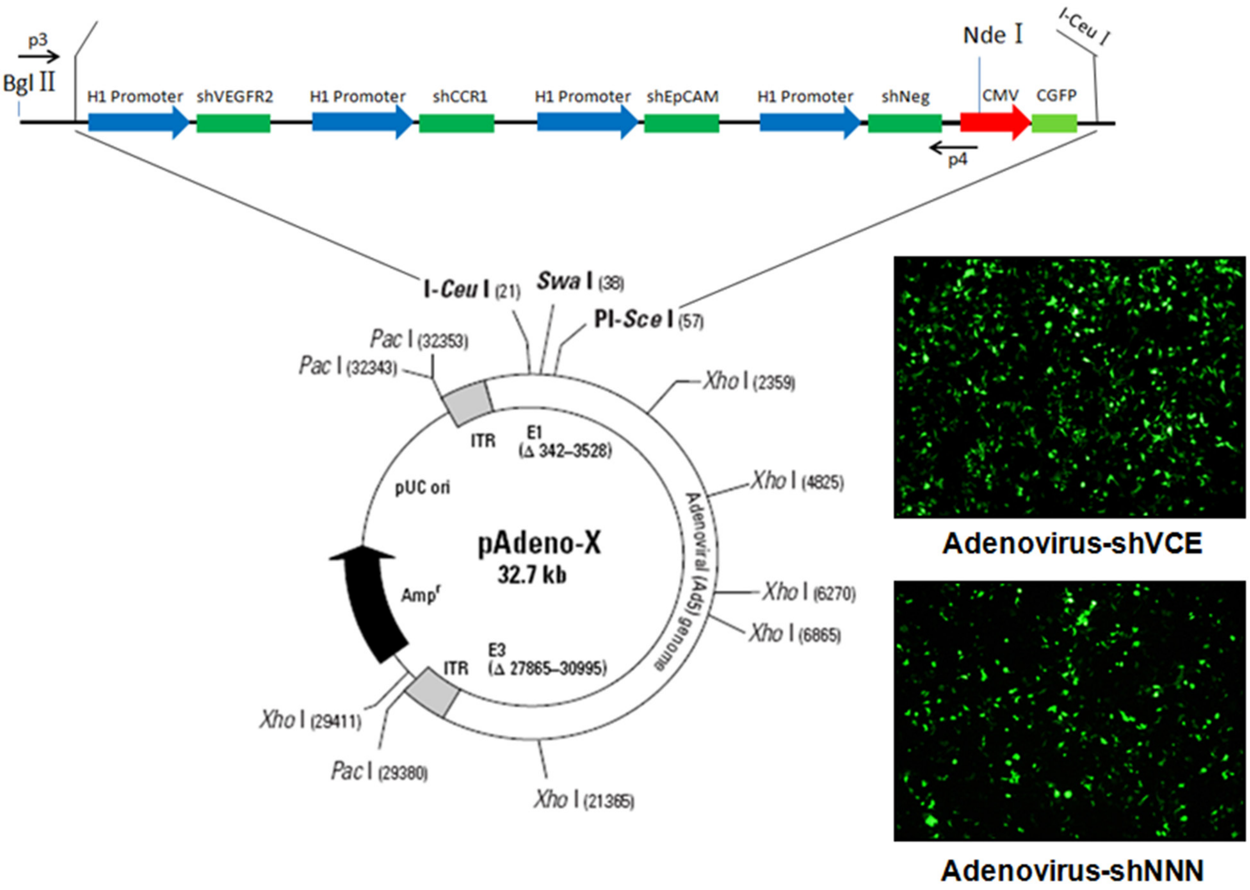

Supplementary Figure S2: Construction of recombinant adenovirus with multiple shRNAs expression. shRNA expression cassettes and CMV promoted cGFP were combined from pRNAT-H1.1 framework, with PI-Sce I/I-Ceu I digested pAdeno-X, to form multiple shRNA expressing recombinant adenovirus.

Supplementary Table S1: Clinical characters of HCC patients. The largest size of multiple tumors was shown in tumor size.

See Supplementary File 1
