# Supplementary material for: A novel multi-target RNAi adenovirus inhibits hepatoma cell proliferation, migration, and induction of angiogenesis
Source: Oncotarget. 2016 May 21;7(36):57705–13. doi: 10.18632/oncotarget.9531 (PMC5295383; doi:10.18632/oncotarget.9531)
Supplement: Supplementary file 2 [file oncotarget-07-57705-s002.doc]

**Supporting Table 1. Clinical characters of HCC patients. The largest size of multiple tumors was shown in tumor size.**

| **Patient number** | **Sex** | **Age** | **HBsAg** | **VEGFR2** | **CCR1** | **EpCAM** | **AFP(ng/ml)** | **Tumor size** | **Tumor nodules** | **Vascular invasion** |
| --- | --- | --- | --- | --- | --- | --- | --- | --- | --- | --- |
| **1** | **M** | **57** | **+** | **+** | **+** | **+** | **10253** | **12*10** | **1** | **-** |
| **2** | **M** | **49** | **-** | **+** | **+** | **+** | **5.39** | **1*1** | **2** | **-** |
| **3** | **M** | **49** | **+** | **+** | **+** | **+** | **698** | **12*10** | **3** | **-** |
| **4** | **M** | **57** | **+** | **+** | **-** | **-** | **317** | **3*3** | **1** | **-** |
| **5** | **M** | **49** | **+** | **+** | **+** | **+** | **6151** | **10*9** | **1** | **-** |
| **6** | **F** | **59** | **+** | **+** | **+** | **-** | **25.99** | **4.5*4.5** | **1** | **+** |
| **7** | **M** | **69** | **+** | **+** | **-** | **-** | **3.72** | **4*3.5** | **1** | **-** |
| **8** | **F** | **49** | **+** | **+** | **+** | **+** | **5.86** | **4*5** | **1** | **-** |
| **9** | **M** | **47** | **+** | **+** | **+** | **+** | **35.08** | **3*4** | **1** | **-** |
| **10** | **M** | **61** | **-** | **+** | **+** | **+** | **264** | **15*18** | **1** | **-** |
| **11** | **M** | **51** | **+** | **+** | **-** | **-** | **7.45** | **9*5** | **2** | **-** |
| **12** | **M** | **61** | **+** | **-** | **-** | **+** | **30.21** | **5*5** | **4** | **-** |
| **13** | **F** | **56** | **-** | **+** | **+** | **+** | **1210** | **4*3** | **1** | **-** |
| **14** | **M** | **69** | **-** | **+** | **+** | **+** | **4.74** | **7*7** | **1** | **-** |
| **15** | **M** | **50** | **+** | **+** | **+** | **+** | **205.4** | **4*5** | **1** | **-** |
| **16** | **M** | **34** | **+** | **+** | **+** | **+** | **22.95** | **3*3** | **1** | **+** |
| **17** | **M** | **65** | **+** | **+** | **-** | **-** | **4.79** | **3.6*3** | **1** | **-** |
| **18** | **M** | **48** | **+** | **+** | **+** | **+** | **28.18** | **3*2.5** | **1** | **-** |
| **19** | **M** | **18** | **+** | **+** | **-** | **+** | **9.92** | **6*5** | **4** | **-** |
| **20** | **F** | **49** | **+** | **+** | **+** | **+** | **366.52** | **6*4.5** | **1** | **-** |
| **21** | **M** | **70** | **+** | **+** | **+** | **+** | **17.61** | **15*13** | **1** | **-** |
| **22** | **M** | **62** | **+** | **+** | **+** | **+** | **3.88** | **8*6** | **1** | **-** |
| **23** | **M** | **42** | **-** | **+** | **+** | **+** | **6793** | **10.5*9** | **1** | **-** |
| **24** | **M** | **38** | **-** | **+** | **+** | **+** | **155.5** | **3.5*3** | **1** | **-** |
| **25** | **M** | **68** | **-** | **+** | **+** | **+** | **41.03** | **13*9** | **1** | **-** |
| **26** | **F** | **72** | **+** | **+** | **+** | **-** | **3.08** | **3*3** | **1** | **-** |
| **27** | **M** | **64** | **+** | **+** | **+** | **+** | **64.76** | **3*4** | **4** | **+** |
| **28** | **M** | **39** | **+** | **+** | **+** | **+** | **632** | **5*5** | **1** | **-** |
| **29** | **M** | **50** | **+** | **+** | **-** | **+** | **1210** | **3*3** | **3** | **-** |
| **30** | **M** | **48** | **+** | **-** | **+** | **-** | **5.91** | **4*2.5** | **1** | **-** |
| **31** | **M** | **42** | **+** | **+** | **+** | **+** | **570.9** | **10.10** | **1** | **-** |
| **32** | **M** | **67** | **+** | **+** | **+** | **+** | **1.50** | **7*5** | **1** | **-** |
| **33** | **F** | **77** | **+** | **+** | **-** | **+** | **3.29** | **7*7** | **1** | **-** |
| **34** | **F** | **58** | **+** | **+** | **+** | **+** | **196.1** | **15*13** | **1** | **-** |
| **35** | **F** | **58** | **+** | **+** | **+** | **+** | **3.75** | **5*4** | **1** | **-** |
| **36** | **M** | **48** | **-** | **+** | **-** | **+** | **187.25** | **6*6** | **1** | **-** |
| **37** | **M** | **29** | **+** | **+** | **+** | **+** | **1.64** | **2*1.5** | **1** | **-** |
| **38** | **M** | **41** | **+** | **+** | **+** | **+** | **9.51** | **6*6** | **3** | **-** |
| **39** | **M** | **48** | **+** | **+** | **+** | **+** | **3.16** | **3*2** | **1** | **+** |
| **40** | **M** | **40** | **+** | **+** | **+** | **+** | **275.8** | **2*2** | **1** | **-** |
